# Biliary Calculi

**Published:** 1870-06

**Authors:** 


Article IV.—*Biliary Calculi.*

In the November No. of this Journal (for 1869) I had the pleasure to lay before its readers a report of a case of removal of these troublesome concretions by the aid of olive oil administered in a large (one pint) dose. Since the date of that communication, the patient has suffered from dyspepsia more or less distressing. Indeed, with a persistence of his old symptoms generally, except the violent pain, until about two weeks ago; at which time he was seized, as before, with the violent, lancinating, cramping pains in the right hypochondrium. Impressed with the efficacy of the olive oil as administered on the former occasion, he procured and drank a pint of oil, upon his own responsibility. There was no alleviation of the symptoms during the succeeding twenty-four hours, at the expiration of which time he passed, per anum, more than one hundred biliary calculi—more than sufficient in bulk to fill a half pint measure.

The case, in addition to the one previously reported, presents no new features, and is only offered as another datum in the therapeusis of this painful malady. W. H.

